# Andrographolide inhibits Burkitt’s lymphoma by binding JUN and CASP3 proteins

**DOI:** 10.1007/s00280-023-04626-4

**Published:** 2023-12-26

**Authors:** Junquan Zeng, Yongliang Zheng, Si Dong, Ting Ding, Shouhua Zhang, Kuangfan Li, Haiyun Liu, Quangang Fang, Sheng Yuan, Yujing Wei, Jing Li, Tingting Liu

**Affiliations:** 1 Department of Hematology, The Affiliated Hospital of Jinggangshan University, Ji’an, 343000 China; 2 First Clinical Medical College, Gannan Medical University, Ganzhou, 341000 China; 3 Department of General Surgery, The Affliated Children’s Hospital of Medical College, Nangchang, 330000 China; 4 Department of Laboratory, Jiangxi Provincial People’s Hospital, The First Affiliated Hospital of Nanchang Medical College, Nangchang, 330000 China; 5 Department of Pathology, Jiangxi Provincial People’s Hospital, The First Affiliated Hospital of Nanchang Medical College, Nangchang, 330000 China; 6 Department of Hematology, Jiangxi Provincial People’s Hospital, The First Affiliated Hospital of Nanchang Medical College, No. 1666, Diezihu Avenue, Nangchang, 330000 Jiangxi China; 7 Department of Drug Discovery and Biomedical Sciences, College of Pharmacy, University of South Carolina, Columbia, SC 29208 USA

**Keywords:** Burkitt’s lymphoma, Andrographolide, Patient-derived xenograft (PDX), JUN (c-Jun), CASP3 (Caspase-3)

## Abstract

**Background:**

Burkitt’s lymphoma, one of the most common subtypes of pediatric malignant lymphoma, is notorious for its swift onset, aggressive proliferation, pronounced invasiveness, and marked malignancy. The therapeutic landscape for Burkitt’s lymphoma currently falls short of providing universally effective and tolerable solutions. Andrographolide, a primary active component of *Andrographis paniculata*, is renowned for its properties of heat-clearing, detoxification, inflammation reduction, and pain relief. It is predominantly used in treating bacterial and viral infections of the upper respiratory tract, as well as dysentery. Various reports highlight the antitumor effects of andrographolide. Yet, its specific impact and the underlying mechanism of action on Burkitt’s lymphoma remain an uncharted area of research.

**Method:**

We employed network pharmacology to pinpoint the targets of andrographolide’s action on Burkitt’s lymphoma and the associated pathways. We then evaluated the impact of andrographolide on Burkitt’s lymphoma using both in vitro and in vivo patient-derived xenograft (PDX) models. Concurrently, we confirmed the molecular targets of andrographolide in Burkitt’s lymphoma through immunofluorescence assays.

**Result:**

Utilizing network pharmacology, we identified 15 relevant targets, 60 interrelationships between these targets, and numerous associated signaling pathways for andrographolide’s action on Burkitt’s lymphoma. In vitro efficacy tests using High-throughput Drug Sensitivity Testing and in vivo PDX model evaluations revealed that andrographolide effectively curtailed the growth of Burkitt’s lymphoma. Moreover, we observed a increased in the expression of JUN (c-Jun) and CASP3 (Caspase 3) proteins in Burkitt’s lymphoma cells treated with andrographolide.

**Conclusion:**

Andrographolide inhibits the growth of Burkitt’s lymphoma by inhibiting JUN and CASP3 proteins.

## Introduction

Burkitt’s lymphoma (BL) is a highly aggressive subtype of B-cell, non-Hodgkin lymphoma, predominantly affecting children and adolescents [1, 2]. It is distinguished by translocation and dysregulation of the proto-oncogene MYC [1]. The World Health Organization (WHO) classifies BL into three subtypes: endemic, sporadic, and immunodeficiency-associated disease [3]. Endemic BL commonly presents in geographical regions where Plasmodium falciparum malaria is endemic. The oncogenic potential of chronic B-cell activation or promotion by Epstein-Barr virus (EBV) has been suggested to contribute to tumor development in cases of malaria co-infection [4]. Immunodeficiency-associated BL is most frequently linked with the Human Immunodeficiency Virus (HIV) [2]. Sustained remission in children and young adults with Burkitt’s lymphoma (BL) is achieved in 60–90% of patients through appropriate treatment, but the overall survival for elderly patients with BL is comparatively lower [5]. The primary treatment regimen for Burkitt’s lymphoma typically involves a combination of cyclophosphamide, vincristine, doxorubicin, methotrexate, ifosfamide, etoposide, and cytarabine (CODOX-M/IVAC), along with rituximab. By employing this treatment approach, 2 year progression-free survival and overall survival (OS) rates were 80% and 84%, respectively, across all patients. For patients classified as low-risk, both rates were 100%, while for high-risk patients, they were 76% and 81%, respectively [6].

As one of the main active components isolated from the herb Andrographolide, andrographolide, a ladanane diterpene, is currently a drug marked in China to treat upper respiratory infections and bacillary dysentery.It has been reported that Andrographolide has a variety of biological effects, including anti-inflammatory, antioxidant, and antitumor properties [7]. Numerous research studies have shed light on the mechanisms underlying the anticancer activity of andrographolide. In breast cancer, andrographolide has been shown to inhibit COX-2 expression by reducing p300 HAT activity and impede angiogenesis via the VEGF pathway [8]; in human colorectal cancer HCT116 cells, andrographolide acts as an antagonist of TNF-α-induced IL-8 production [9]. Moreover, it exhibits the ability to suppress interleukin-6 expression and restrain the growth of prostate cancer cells [10]. In colorectal cancer cells, the anticancer effect of andrographolide has been linked to a GSK-3β-independent Wnt/β-catenin signaling pathway [11].

However, the therapeutic effects and underlying mechanisms of andrographolide on Burkitt’s lymphoma are not yet fully understood. The efficacy and potential adverse reactions of current clinical antitumor drugs pose significant challenges in tumor therapy. Hence, this study aimed to investigate the efficacy and mechanism of andrographolide in the context of Burkitt’s lymphoma. The findings of this research endeavor will contribute to establishing a theoretical foundation for anti-Burkitt’s lymphoma treatment and facilitate the clinical translation of andrographolide as a therapeutic agent.

## Materials and methods

### Collection of andrographolide targets

The PubChem database (https://pubchem.ncbi.nlm.nih.gov/) was utilized to obtain information on paniculta lactone. For target protein identification, the following databases were used: Swiss Target Prediction (http://www.swisstargetprediction.ch/), BATMAN-TCM (http://bionet.ncpsb.org/batman-tcm/), STITCH (http://stitch.embl.de/), and ChEMBL (https://www.ebi.ac.uk/chembl/). The obtained target proteins were cross-referenced and validated using UniProt. In the STITCH database, a threshold of Score > 300 was set for screening target proteins, while in the BATMAN-TCM database, a Cut off > 20 was utilized. In the ChEMBL database, a threshold of score > 5 was employed for target protein screening.

### Burkitt lymphoma gene collection

Burkitt lymphoma related genes were searched in DisGeNET (Score_gda > 0.1), Genecards (Relevance score > 10) and OMIM database.

### Construction of andrographolide: target-Burkitt lymphoma network

To identify potential candidate genes of andrographolide for Burkitt’s lymphoma, the targets of andrographolide were intersected with disease-related genes associated with Burkitt’s lymphoma. The resulting overlapping genes were considered as candidate genes for andrographolide in the context of Burkitt’s lymphoma. Using the Cytoscape software, a network model was constructed to illustrate the relationships among andrographolide, its corresponding targets, and Burkitt’s lymphoma. The network visualization and analysis were performed using the CytoNCA plugin within Cytoscape, which allows for the examination of network topology and the identification of important nodes or genes within the network.

### PPI network construction

The common target dataset was imported into the STRING database (https://string-db.org/) with the species set as *Homo sapiens*. A minimum required interaction score of 0.4 was selected, and the resulting “string_interactions_short-tsv” file was downloaded. This file was then imported into Cytoscape 3.7.2 for network visualization. The CytoNCA plugin was utilized for network topology analysis, focusing on Degree Centrality (DC), Betweenness Centrality (BC), Closeness Centrality (CC), and Eigenvector Centrality (EC) using the Local Average Connections-based method (LAC) and Network Centrality (NC). In the network visualization, nodes with brighter colors and larger sizes represent higher degree values and greater importance within the network. To further analyze the core genes, the “string_interactions_short.tsv” file was imported into R software. The number of connecting nodes for each core gene was obtained, and a bar chart illustrating the top 30 core genes was generated.

### GO and KEGG enrichment analysis

For the gene ontology (GO) and Kyoto Encyclopedia of Genes and Genomes (KEGG) enrichment analysis of the candidate genes of andrographolide for Burkitt’s lymphoma, R-language packages including “ClusterProfiler,” “org.Hs.eg.Db,” and “ggplot2” were utilized. In the enrichment analysis, the adjusted threshold values for the *p* value and *q* value were set to 0.05. This threshold helps identify significantly enriched GO terms and KEGG pathways associated with the candidate genes. The ClusterProfiler package was used to perform the enrichment analysis, while ggplot2 was employed for visualizing the results, allowing for the creation of informative plots representing the enriched GO terms and KEGG pathways related to Burkitt’s lymphoma and the core target genes influenced by andrographolide.

### Molecular docking verification

To assess the affinity of key hubs in the network, molecular docking analysis was employed. The three-dimensional (3D) structure of the ligand (andrographolide) was obtained from the PubChem database, while the three-dimensional structure of the receptor (target protein) was acquired from the RCSB database (http://www.rcsb.org/). Both the ligands and receptors were processed and optimized using the mgltools_win32_1.5.6 software, ensuring proper preparation and compatibility for docking studies. The prepared ligands and receptors were saved as PDBQT files. For the docking calculations, AutoDock Vina 1.1.2 software (http://vina.scripps.edu/) was utilized. This software performed the docking simulations to estimate the affinity of the interactions between the key active ingredient (andrographolide) and the target protein. After the molecular docking analysis, the resulting data was visually represented using Discovery Studio 2016 software, providing graphical visualization of the docking results and allowing for a comprehensive analysis of the binding interactions between andrographolide and the target protein.

### Patient sample materials and PDX establishment

All the patient samples of Burkitt’s lymphoma used in this study were obtained from Jiangxi Children’s Hospital. The study protocol was approved by the Clinical Research Ethics Board of Jiangxi Children’s Hospital, ensuring compliance with ethical guidelines and patient privacy. To establish Burkitt’s lymphoma patient-derived xenografts (PDX), the same methodology as previously reported was employed. The PDX models numbered as No. 1109, No. 378, and No. 63 were generated in collaboration with Nanchang Royo Biotech company, following established procedures [12].

### Other materials

All animal experiments conducted in this study were approved by the Nanchang Royo Biotech Company Laboratory Animal Welfare Ethics Committee (Approval No. RYE2017080101). The welfare and ethical treatment of the animals were ensured throughout the experiments. The mice used in the study were maintained in a specific pathogen-free (SPF) environment to minimize the risk of contamination or external influences. Six-to-eight-week-old NOD-SCID or BALB/c nude mice, known for their immunodeficiency, were selected as appropriate models for the experiments. These mice were chosen due to their suitability for studying Burkitt’s lymphoma and to ensure consistency with established research protocols.

### Hydrogel-embedded histoculture drug sensitivity test (HDST) in vitro

Following the previously reported protocol [12], the PDX tissues were dissected into small fragments measuring 3 mm × 3 mm × 3 mm. These fragments were then treated with different concentrations of Andrographolide, including 0.25, 0.5, 1, 2, 4, 8, and 16 μM. Each treatment was performed using 700 μL of tissue culture medium. After incubating the PDX fragments with Andrographolide for 3 days, the supernatant was replaced with fresh medium containing an equal amount of the drug. Following a total treatment duration of 5 days, the viability of the cells was assessed using either the CCK8 assay or the Alamar Blue assay. These assays are commonly employed to evaluate cell viability and determine the impact of drug treatment on cell survival and proliferation.

### Drug sensitivity test in PDX model

When the volume of Burkitt’s lymphoma PDX tumors reached 40–100 mm^3^, the PDX mice were divided into two groups. One group received treatment with Andrographolide at a dosage of 25 mg/kg, administered intraperitoneally (i.p.) once daily, while the other group received saline as a control, also at a dosage of 25 mg/kg i.p. once daily. The treatment was continued for a period of 3–5 weeks or until the tumor volume reached or exceeded 2000 mm^3^. The tumor volumes of the Andrographolide-treated group were compared with those of the control group throughout the treatment period. Tumor growth inhibition (TGI) was calculated using the formula: (1 − *T*/*C*) * 100%, where *T* represents the average tumor weight of the treatment group and *C* represents the average tumor weight of the control group. TGI provides a measure of the relative reduction in tumor growth due to Andrographolide treatment compared to the control group.

### Immunofluorescence (IF)

The samples were initially fixed with paraformaldehyde (PFA), followed by embedding in paraffin and slicing into 4 μm sections. These sections were subsequently blocked using 5% goat serum and 0.3% Triton X-100 (Sigma) in TBS buffer, a process which lasted for 45 min. After the blocking, the sections were exposed to the primary antibody for JUN (1:200, Servicebio, Catalogue # GB11071) and CASP3 (1:200, Servicebio, Catalogue # GB11532) and left to incubate overnight at room temperature. The sections were then rinsed three times, with each rinse lasting a minimum of 30 min, using PBS buffer. The final step involved the incubation of the sections for 90 min at room temperature with secondary antibodies (1:500, Alexa Fluor 568, Alexa Fluor 488, Life Technologies).

## Result

### Construction of target network between andrographolide and Burkitt’s lymphoma

**Fig. 1:**
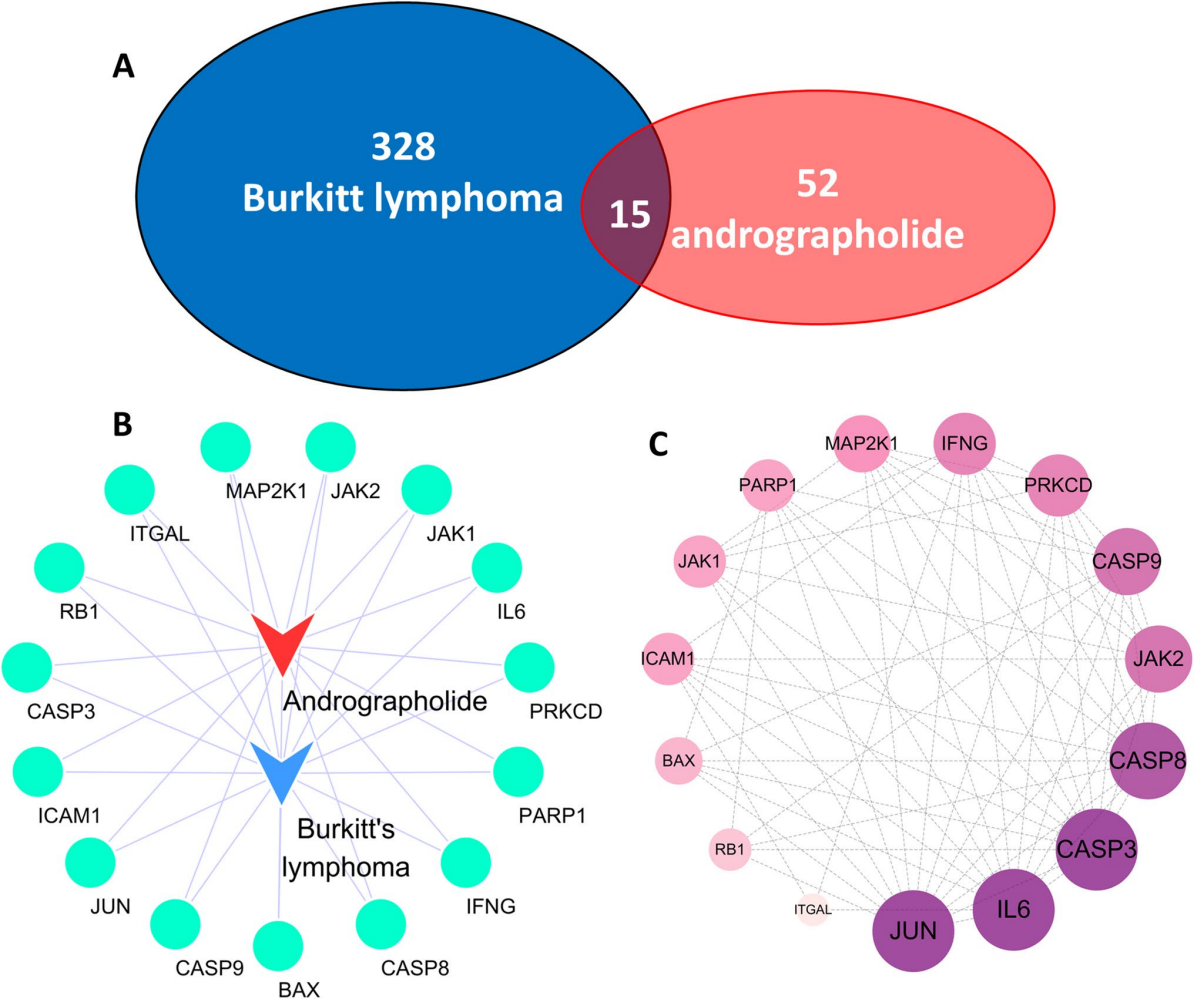
The target of andrographolide in the treatment of Burkitt lymphoma was analyzed by network pharmacology. **A** Intersection gene of Burkitt lymphoma and andrographolide action. **B** Intersection gene of Burkitt lymphoma and andrographolide action. **C** PPI network diagram of the target

Fifty targets associated with andrographolide were predicted using its structural data and the Swiss Target Prediction tool. However, Batmant-Tcm didn’t predict any targets, while STITCH predicted 20. The ChEMBL database also failed to predict any targets. After collating and harmonizing the data, we identified 68 genes linked to andrographolide. In the second step, we sourced 556 Burkitt-related genes from the DisGeNET database, among which 57 met our screening criteria. The Genecards database provided 1400 genes related to Burkitt’s lymphoma, with 305 satisfying the screening criteria. The OMIM database, on the other hand, listed 34 genes associated with Burkitt lymphoma. After eliminating duplicates, we collected a total of 343 unique Burkitt lymphoma genes. Upon intersecting the andrographolide-related genes and Burkitt’s lymphoma disease targets, we identified 15 shared genes (Fig. 1A). These shared genes, along with andrographolide, were used to create a “andrographolide-target-Burkitt’s lymphoma” network comprising 30 nodes and 30 relationships (Fig. 1B). When the common target dataset was introduced into the STRING database, a protein interaction network was formed. The PPI network’s tsv files were then imported into Cytoscape, revealing 15 related targets and 60 mutual relationships among them. According to the ranking of degree values, the obtained targets included CASP3 [13], IL6 [13], JUN [13], CASP8 [12], CASP9 [10], JAK2 [10], and others (Fig. 1C).

### GO and KEGG enrichment analysis of andrographolide treatment for Burkitt’s lymphoma

**Fig. 2:**
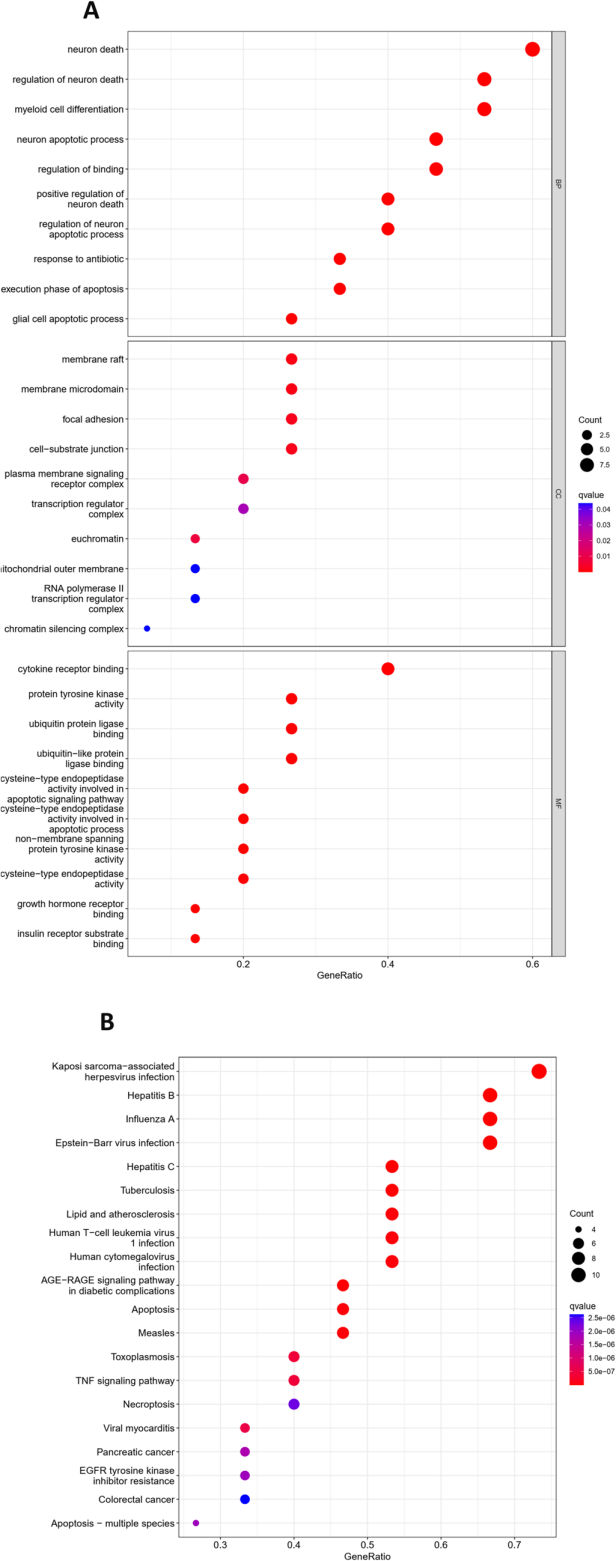
Andrographolide signaling pathway in the treatment of Burkitt lymphoma. **A** GO enrichment analysis. **B** KEGG enrichment analysis

Andrographolide’s therapeutic role in Burkitt’s lymphoma involves participation in 1029 biological processes, including neuron death, regulation of neuron death, positive regulation of neuron death, myeloid cell differentiation, and response to neuron death antibiotic, among others. In terms of cellular components, it interacts with 12 constituents such as the membrane raft, membrane microdomain, focal adhesion, cell-substrate junction, and euchromatin, to name a few. Andrographolide also exhibits 62 molecular functions. These encompass cytokine receptor binding, cysteine-type endopeptidase activity involved in the apoptotic signaling pathway, cysteine-type endopeptidase activity engaged in the apoptotic process, protein tyrosine kinase activity, and non-membrane spanning protein tyrosine kinase activity, among others (Fig. 2A). The treatment also implicates 108 pathways, including apoptosis, TNF signaling pathway, apoptosis—multiple species, necroptosis, colorectal cancer, and other signaling pathways (Fig. 2B).

### Validation of molecular docking between andrographolide and Burkitt’s lymphoma

**Fig. 3:**
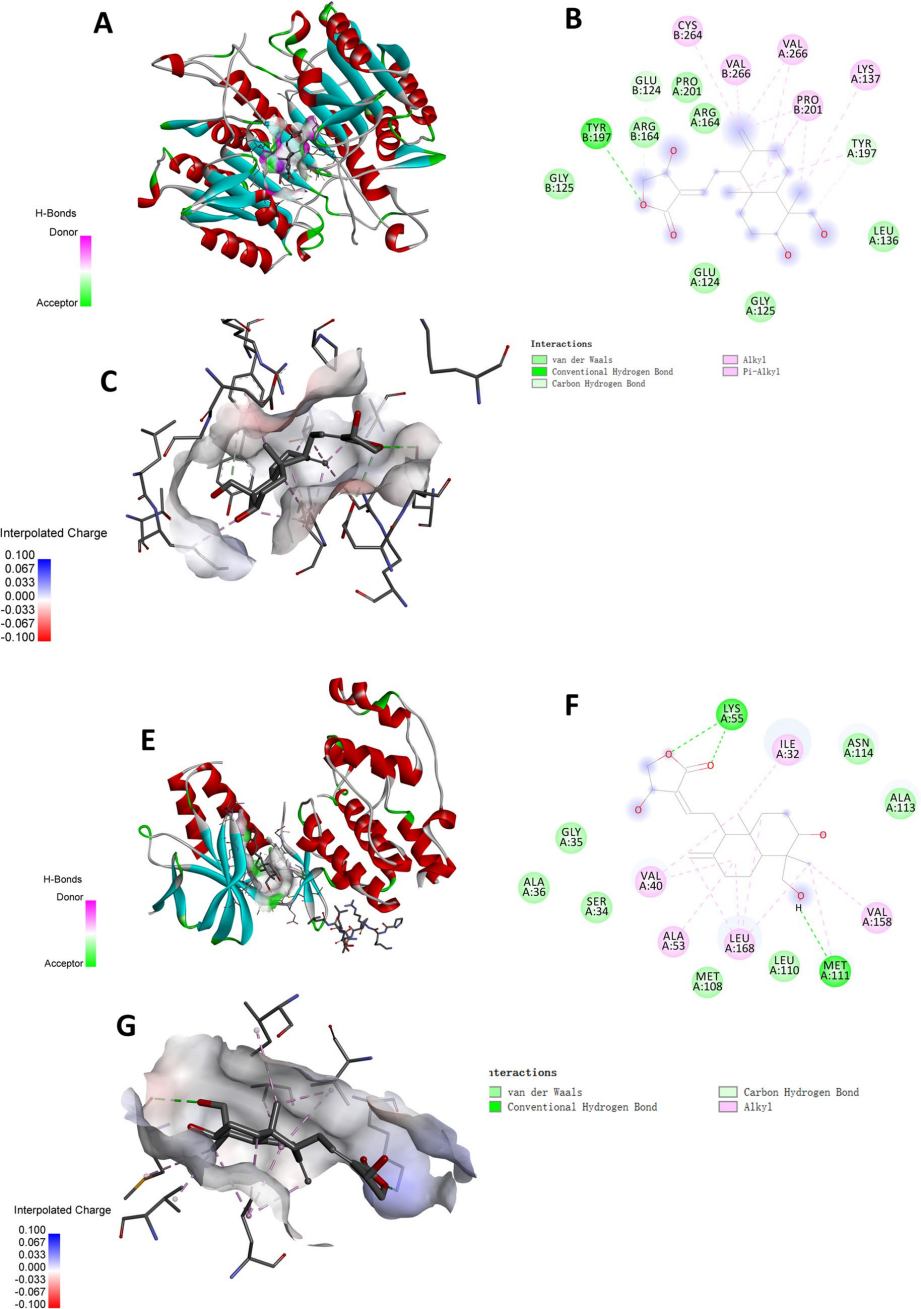
Molecular docking diagram of andrographolide and target. **A–C** Docking diagram of andrographolide and JUN protein. **D–F** Docking diagram of andrographolide and CASP3 protein

Andrographolide was subjected to molecular docking with three core targets—JUN, IL6, and CASP3. As a rule of thumb, efficient binding energy is less than − 7 kcal/mol. According to our results, JUN demonstrated the highest affinity to andrographolide with a binding energy of − 7.4 kcal/mol, closely followed by Caspase-3 (CASP3) which exhibited a binding energy of − 7.3 kcal/mol. IL6, on the other hand, displayed relatively lower affinity with a binding energy of − 6.8 kcal/mol, exceeding the − 7 kcal/mol threshold (Table 1). Upon analyzing the docking conformation of andrographolide with the JUN protein (Fig. 3A), we observed that andrographolide is situated within the JUN protein band. This small molecule establishes hydrogen bonds with the amino acids MET111 and LYS55, and engages in hydrophobic interactions with amino acids such as ASN114, ALA113, LEU110, MET108, and others. Moreover, alkyl bonds were formed with ILE32, VAL158, LEU168, among others. In the case of the andrographolide-CASP3 protein docking diagram (Fig. 3B), andrographolide was found within the CASP3 protein band. Here, hydrogen bonding occurred with the amino acid TYR197, and hydrophobic interactions were evident with amino acids including PRO201, GLU124, GLY125, and so forth. Furthermore, alkyl interactions were formed with LYS137, PRO201, and VAL266.

**Table 1:** Target information and binding energy

GENE	PDB	*X*	*Y*	*Z*	Affinity (kcal/mol)
JUN	4IZY	− 12.961	6.374727	− 21.2683	− 7.4
IL6	1IL6	18.992	1.55	2.023	− 6.8
CASP3	3H0E	24.89247	53.58088	12.29375	− 7.3

The PDB is the number of proteins resolved by different researchers in the RCSB database. *XYZ* is the central coordinate of the protein binding site, and affinity (binding energy) is the standard for Autodock to evaluate the interaction between proteins and small molecules. Generally, binding energy less than − 7 indicates strong binding ability between proteins and small molecules

### Andrographolide inhibited the growth of Burkitt’s lymphoma in vivo and in vitro

**Fig. 4:**
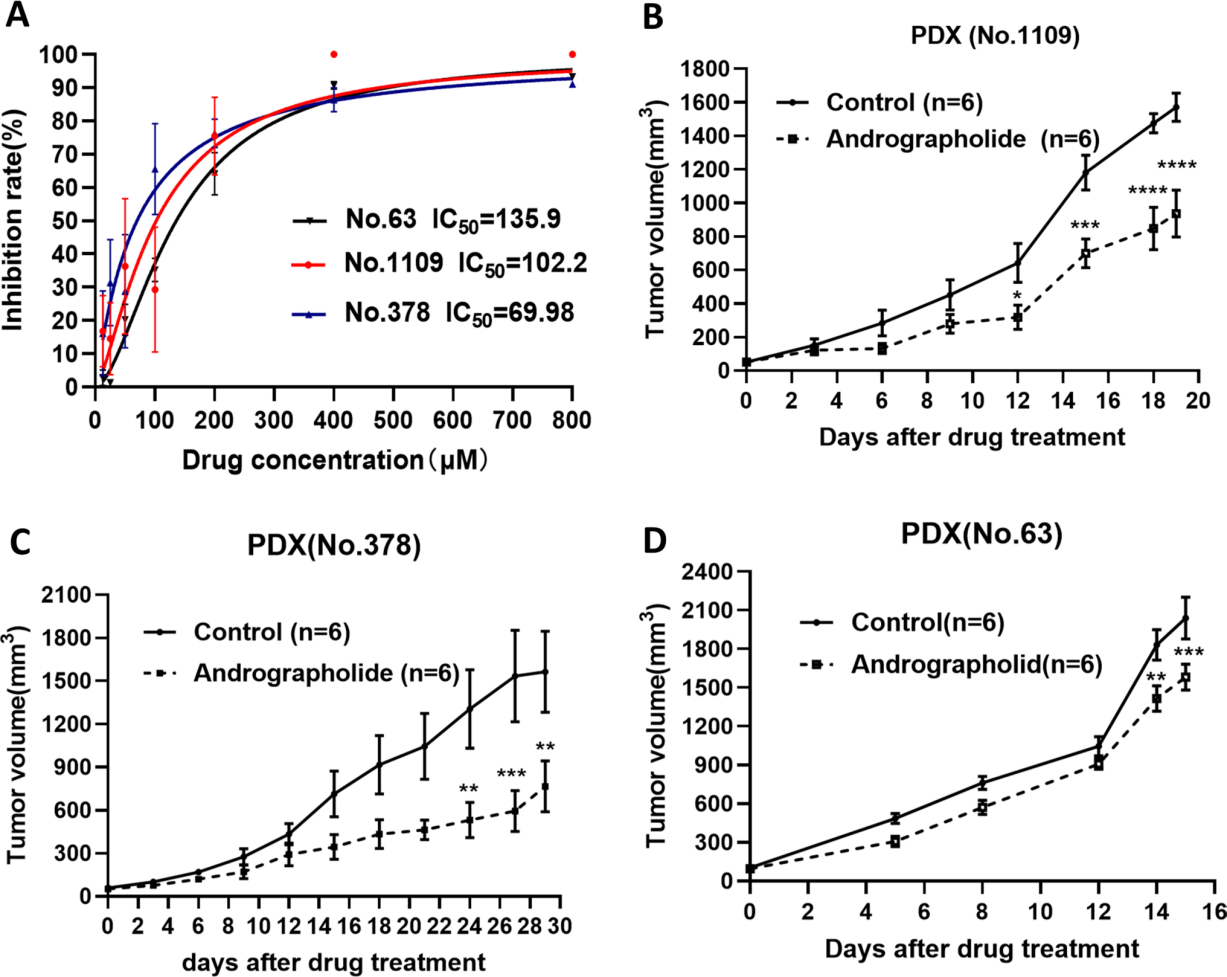
Andrographolide inhibited the growth of Burkitt’s lymphoma in vitro and in vivo. **A** Dose–effect curve of andrographolide on Burkitt lymphoma by HDST. **B** Andrographolide inhibits the growth of Burkitt lymphoma in PDX mice

To verify the effect of andrographolide on Burkitt’s lymphoma, we investigated its in vitro efficacy on the disease via High-Definition Single-Cell Assay (HDST), and in vivo effectiveness through drug sensitivity testing in Patient-Derived Xenograft (PDX) mice. The dose–response curve from the HDST result indicated that andrographolide significantly inhibited the proliferation of lymphoma cells (IC_50_ of No. 1109 = 102.02; IC_50_ of No. 378 = 69.98; IC_50_ of No. 63 = 135.9) (Fig. 4A). In terms of in vivo efficacy, andrographolide exhibited a demonstrable suppression of Burkitt’s lymphoma growth in patient-derived xenograft (PDX) mice (Fig. 4B–E).

### Andrographolide increased JUN and CASP3 protein expression in Burkitt’s lymphoma PDX

**Fig. 5:**
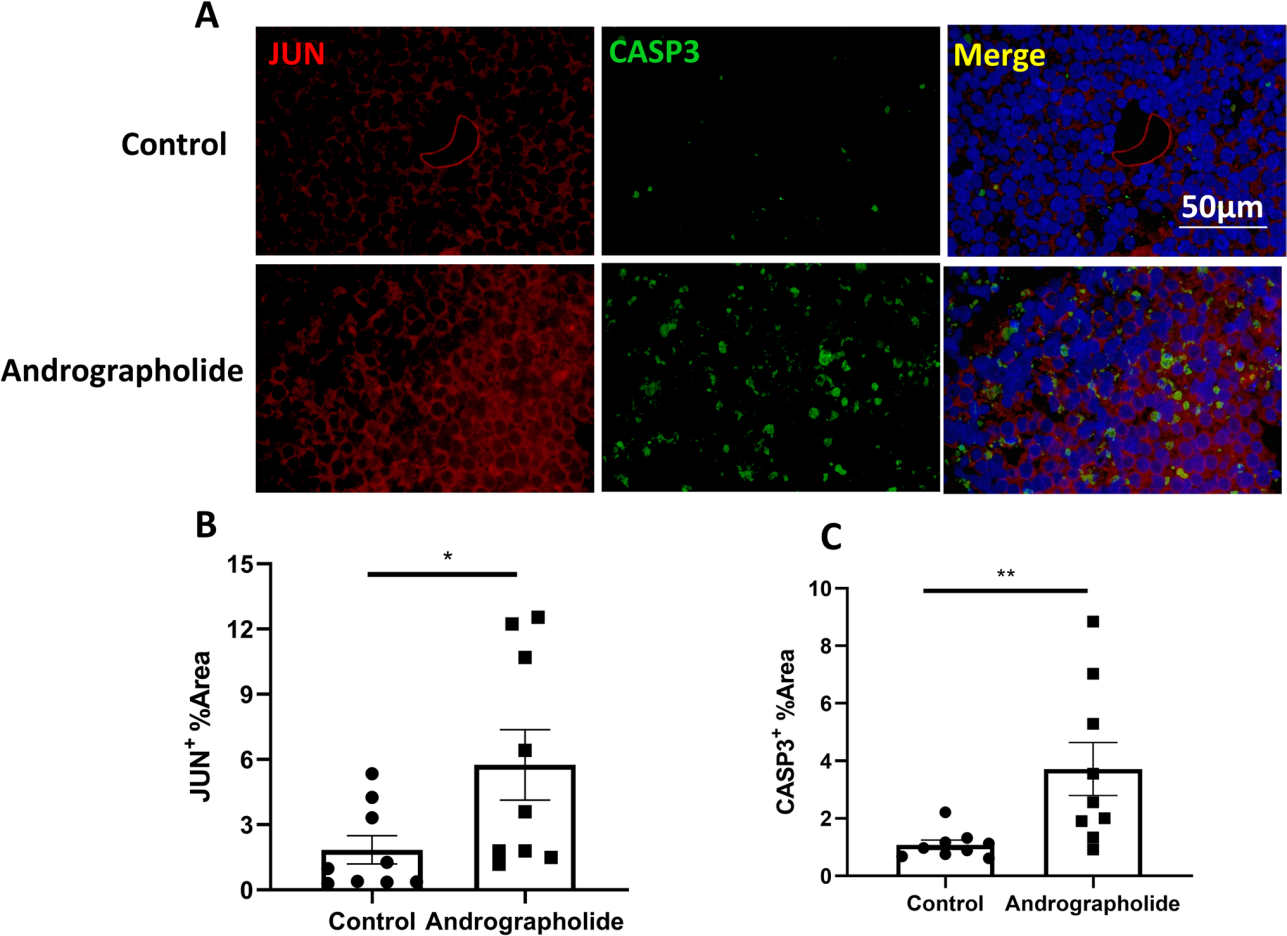
Andrographolide increased JUN and CASP3 protein expression in Burkitt’s lymphoma PDX. **A** Expression of JUN and CASP3 proteins in andrographolide treated Burkitt lymphoma. **B**, **C** Expression statistics of JUN and CASP3 proteins

To verify the molecular mechanism of andrographolide inhibits Burkitt’s lymphoma, we examined JUN and CASP3 protein expression in andrographolide treated Burkitt’s lymphoma by IF. The results showed that andrographolide increased JUN and CASP3 protein expression in Burkitt’s lymphoma PDX (Fig. 5).

## Discussion

This study discovered that andrographolide inhibited the progression of Burkitt’s lymphoma by interacting with JUN and CASP3 proteins. JUN operates as an oncogenic transcription factor, responsible for the coding of the protein c-Jun, which is integral to cell cycle progression, overseeing the regulation of cyclin D1 and p53 [13–15]. In addition, it assumes an anti-apoptotic role in numerous tumors [16]. Previous research reported that andrographolide induced G2/M arrest and prompted cell apoptosis by regulating the c-Jun N-terminal kinase (JNK) pathway in osteosarcoma cells [17]. In the context of Burkitt’s lymphoma, JUN remains a crucial protein for andrographolide, potentially serving as a marker protein for the therapeutic application of andrographolide in treating Burkitt’s lymphoma. It was reported that apoptosis was mediated through mitochondrial pathways [18]. Moreover, as a principal instigator of apoptosis, induction of CASP3 by a wide range of anticancer therapies, including cytotoxic drugs, radiotherapy, or immunotherapy, can trigger tumor cell death [19]. As such, it is commonly used as an efficacy marker in cancer therapy [20]. Our result showed that andrographolide could increased CASP3 expression, increased apoptosis, and which made a negative feedback regulation to increases the expression of JUN protein.

Patient-derived xenograft (PDX) models, generated by implanting human tissues (tumor, enriched circulating tumor cells) into immunodeficient mice, retain many key characteristics of patients’ tumors including histology, genomic signature, cellular heterogeneity, and drug responsiveness, and the original tumor microenvironment [21, 22]. PDX models. DST method is an in vitro detection method matching the pharmacodynamic evaluation of PDX. Using HDST method is faster, simpler, and cheaper than using PDX model. The two methods together can verify the effect of the drug on each other. We tested the in vitro and vivo efficacy of andrographolide in three cases of Burkitt’s lymphoma with PDX. The effect of andrographolide on Burkitt’s lymphoma is in accordance with each other. Although andrographolide inhibited Burkitt lymphoma, the degree of inhibition was different (the IC_50_ of No. 63 > the IC_50_ of No. 1109 > the IC_50_ of No. 378; the TGI of No. 378 > the TGI of No. 1109 > the TGI of No. 63). There are significant individual differences among Burkitt’s lymphomas that affect the effectiveness of andrographolide. We observed that JUN and CASP3 expression were lower in Burkitt lymphoma instances where andrographolide inhibition was weak compared to those with strong inhibition. Thus, JUN and CASP3 expression might be employed as predictive markers for andrographolide in the treatment of Burkitt’s lymphoma. Andrographolide is the most widely used monomer in Chinese medicine. According to its medicinal guidelines, it is associated with fewer adverse reactions. Our research verifies the inhibitory action of andrographolide on Burkitt’s lymphoma, which could facilitate its clinical application. Andrographolide can be used either as a standalone treatment or in combination with other therapies for Burkitt’s lymphoma, presenting minimal adverse effects. This makes it a potentially beneficial candidate for expanding treatment options in Burkitt’s lymphoma management.

## Conclusion

In this paper, we used the method of network pharmacology to obtain the target and related pathway of andrographolide inhibiting Burkitt’s lymphoma. And the inhibition of andrographolide on Burkitt’s lymphoma was verified by PDX model by JUN and CASP3 protein.

## Data Availability

The datasets used and/or analyzed during the current study are available from the corresponding author on reasonable request.
